# Abortion beyond 13 weeks in Argentina: healthcare seeking experiences during self-managed abortion accompanied by the *Socorristas en Red*

**DOI:** 10.1186/s12978-022-01488-6

**Published:** 2022-08-26

**Authors:** Brianna Keefe-Oates, Chelsea G. Tejada, Ruth Zurbriggen, Belén Grosso, Caitlin Gerdts

**Affiliations:** 1 Ibis Reproductive Health, 2067 Massachusetts Ave, Suite 320, Cambridge, MA 02140 USA; 2 La Colectiva Feminista La Revuelta, Neuquen, Argentina; 3 Ibis Reproductive Health, Oakland, CA USA; 4 Present Address: Department of Social and Behavioral Sciences, Harvard University TH Chan School of Public Health, Boston, USA

**Keywords:** Self-managed abortion, Reproductive health, Argentina, Activism, Health services

## Abstract

**Background:**

In Argentina, a group of feminist activists, the *Socorristas en Red*, provide information and accompaniment to people seeking abortions, including beyond 13 weeks gestation. Recently-released WHO guidelines for abortion care acknowledge that abortion trajectories vary and people may seek services and support from a range of settings in the process of an abortion. It follows, therefore, that people who self manage abortions beyond 13 weeks with the support of accompaniment groups may interact with health professionals in the public and/or private sector. Understanding the reasons for and experiences with these interactions can help to inform best practice.

**Methods:**

In 2016, we conducted 23 exploratory interviews among women who self managed abortions beyond 13 weeks gestation accompanied by *Socorristas,* to understand healthcare-seeking decisions and experiences. We used narrative inquiry as an interview technique and coded interviews using first a holistic coding and, second, a content analysis technique to identify emergent themes in the text and subsequently identify themes relevant to study aims.

**Results:**

We found that many participants had disclosed their abortion intentions to health professionals prior to their abortions. Some were provided with emotional support and referrals to the *Socorristas*, while others were admonished and warned of serious health consequences. Most participants sought post-abortion care in public or private-sector health facilities; for fear of legal repercussions, many participants did not share that they had used abortion medications with post-abortion care providers. During care seeking, some participants reported poor treatment, in several cases because they were suspected of inducing abortion, while others reported supportive care from health professionals who had previously-established relationships with the *Socorristas*.

**Conclusions:**

This study illuminates the important role that supportive health professionals can play to ensure that, regardless of the trajectory of an abortion, people feel comfortable accessing clinical services during their abortion process, even in restrictive settings. Feminist activists can help build bridges with the medical system to ensure that providers who interact with people seeking abortion-related services are empathic, understand their legal rights, and provide supportive care.

## Plain language summary

Before 2021, Argentina’s laws prohibited abortion except in limited circumstances. A feminist activist group, the *Socorristas en Red,* provided information and support to people seeking abortion services, including those beyond 13 weeks gestation. Recently-released WHO guidelines for abortion care acknowledge that abortion trajectories vary and people may seek services and support from a range of settings in the process of a single abortion (World Health Organization, 2022). It follows, therefore, that people who self manage abortions beyond 13 weeks with the support of accompaniment groups may interact with health professionals in the public and/or private sector. Understanding reasons for and experiences with these interactions can help improve quality of abortion care and inform best practice. We conducted interviews with people who used medications for abortion in the second trimester supported by the *Socorristas* and analyzed their stories about abortion-related healthcare. Participants in our study sought healthcare services in clinics at various timepoints: many visited providers when first considering abortion, and most went to providers for post-abortion care. Some providers admonished their patients for seeking abortion services, while others provided referrals to the *Socorristas*. Many participants were concerned about their legal safety, and feared prosecution when they received healthcare services, especially if seeking care after the abortion. In these instances, some participants experienced poor quality or inappropriate care because they did not feel safe sharing that they had taken medications for abortion, and others were treated poorly because of the abortion. The experiences of those who sought services from health professionals who had established relationships with the *Socorristas* appeared to be more positive than the others. This study illuminates the important role that supportive health professionals can play to ensure that, regardless of the trajectory of an abortion, people feel comfortable accessing clinical services during the course of an abortion, even in restrictive settings, and how feminist activists can help to ensure providers are empathic and provide supportive care to all those who have abortions.

## Abstract (En Español)

**Introducción:**

En Argentina, una red de colectivas feministas, las Socorristas en Red, brindan información y acompañamiento a las personas que buscan abortos, incluso más allá de las 13 semanas de gestación. La nueva guía de la OMS para la atención en el aborto reconoce que las trayectorias del aborto varían y las personas pueden buscar servicios y apoyo en una variedad de entornos en el proceso de un aborto. Las personas que autogestionan abortos más allá de las 13 semanas con el apoyo de grupos de acompañamiento podrán interactuar con profesionales del sector médico. Comprender las razones y las experiencias con estas interacciones puede ayudar a mejorar las prácticas.

**Métodos:**

Realizamos 23 entrevistas en 2016 con mujeres que tuvieron abortos después de las 13 semanas usando medicamentos fuera de un entorno clínico acompañadas por Socorristas, para comprender las decisiones y experiencias con el sistema de salud. Utilizamos la indagación narrativa como técnica de entrevista y codificamos entrevistas utilizando las técnicas de codificación holística y análisis de contenido para identificar los temas emergentes en el texto y luego identificar temas relevantes a los objetivos del estudio.

**Resultados:**

Antes del aborto, muchas mujeres revelaron sus intenciones a los profesionales de la salud; algunos brindaron apoyo emocional y referencias a las Socorristas, y otros las amonestaron y advirtieron sobre consecuencias para la salud. La mayoría de las mujeres buscaron atención postaborto; muchas temían repercusiones legales al buscar atención y no dijeron que habían usado medicamentos. Durante la búsqueda de atención, algunas mujeres reportaron mal trato, en varios casos porque se sospechaba que inducían el aborto, mientras que otras reportaron apoyo por parte de profesionales de la salud que colaboraban previamente con las Socorristas.

**Conclusiones:**

Este estudio demuestra cómo los profesionales de la salud que brindan apoyo pueden ayudar a garantizar que las personas que buscan servicios de aborto se sientan cómodas al acceder a los servicios clínicos en entornos restrictivos. Las activistas feministas pueden ayudar a construir puentes con el sistema de salud para garantizar que los profesionales de salud sean empáticas, respetan los derechos legales y brinden atención de apoyo.

## Introduction

The use of misoprostol alone or combined with mifepristone (referred to as medication abortion) is a World Health Organization (WHO)-recommended method for abortion throughout pregnancy [1, 2]. A robust body of evidence has demonstrated the safety of medication abortion when provided through a variety of out-of-clinic settings including telemedicine, harm reduction, and support through feminist hotlines and lay health workers and activists [3–13]. A recent study established that self-managed medication abortion (defined as ‘the use of medications to end a pregnancy on one's own, without clinical supervision’) with support from feminist accompaniment groups is no less effective than clinician-managed medication abortion [14, 15]. Newly-released WHO guidelines on abortion care now recognize the self-use of medications for abortion as one of a range of safe and effective models of care [1].

For people seeking abortions in legally restrictive settings where legal, financial, logistical or other barriers prevent or deter them from obtaining a clinic-based abortion, out-of-clinic models of abortion care can provide a safe and supportive option. In these contexts, barriers to access—such as a lack of trained or willing providers, stigma, and fear of legal consequences—increase delays for those seeking abortion, thereby increasing the proportion of abortions that happen later in pregnancy [16–20]. These same people are often those who experience marginalization—globally studies have found that those seeking care beyond 13 weeks are more likely to be younger and live in poverty [18, 20]. While abortion at any gestation is safer than childbirth, the risks associated with abortion do increase with gestational age, and where abortions at a later gestation are harder to access, some may resort to unsafe methods while others may be forced to carry unwanted pregnancies to term [16, 21–23].

In many settings where abortion is legally restricted, or where barriers to abortion access persist, safe abortion hotlines and feminist activists have created models to provide those seeking abortion with information on how to safely self-manage abortion using WHO-recommended medications [24]. One such model is that of *accompaniment*, where feminist activists support people, either in person or over the phone, with information and emotional support during their abortion [9, 15, 24–27]. In these cases, the activists have an initial contact with an individual to provide them information about the medication and emotional support when an individual might need it. They are then in touch with the individual throughout their abortion process, most commonly via phone but occasionally in person, to provide them the support they need to ensure they have a safe, high-quality abortion [25, 27–29].

Some, but not all, of these models support people seeking abortion after 13 weeks [9, 25, 30]. Data on these experiences are sparse, however two retrospective chart reviews showed high rates of abortion completion among those accompanied after 13 weeks, similar to rates of completion in clinic-based studies, and high rates of satisfaction with the experience of being accompanied later in pregnancy have also been reported [12, 31, 32]. Two qualitative studies documenting activists’ experiences providing accompaniment for medication abortion beyond 13 weeks in Latin America found that activists had to be more attentive to people’s needs compared to a first-trimester abortion because the process took longer, and because there were more legal and medical risks involved [25, 29].

People who self-manage abortions with accompaniment support may also encounter health professionals in clinical health systems at various points in their abortion trajectory, including when they find out they are pregnant, for an ultrasound prior to the abortion, to check on any warning signs of complications, or when seeking post-abortion care such as contraceptives or confirmation of abortion completion. Access to a clinic-based healthcare provider, even for those who would prefer to self-manage an abortion, is a key tenet of quality of abortion care as defined by the WHO for abortion at any gestation, and can be important for many reasons, especially for abortions that happen later in pregnancy when the small risk of complications does increase with gestation [1, 33, 34]. Yet healthcare provider interactions are not without risk—especially in contexts where abortion is legally restricted, and individuals may risk being shamed, reported, or even prosecuted for attempting an abortion [35, 36]. In order to inform best practices and for health services to implement the standards established in the new WHO guidelines it is critical that all people have access to compassionate, supportive, and safe health care throughout the trajectory of their abortion, regardless of whether the abortion is self-managed or clinician-managed. Understanding the reasons for healthcare seeking in healthcare systems among those who self-manage abortions beyond 13 weeks, as well as people’s experiences receiving those healthcare services, can help to improve quality of abortion care and inform best practices.

In Argentina, abortion was only legal in cases of rape or when the ‘woman’s’ health or life was at risk until 2021. In December 2020, the Argentinean Congress legalized abortion in all cases through 14 weeks, however abortion continues to be legal past that point only under the indications already established [37]. Prior to this legalization of early abortion care, though maternal deaths were rare in the country, 17% of those were attributed to unsafe abortion between 2014–2016; more recent data are unavailable [38]. To prevent maternal morbidity and mortality, post-abortion services have historically been legal in Argentina; providers are required, through the professional code of ethics, to care for those who have had abortions, theoretically without judgement or reporting an abortion outside of the legal regulations to the authorities [39]. However, previous research has shown that the quality of those services varied widely by provider and facility, and fears of being judged or reported to the authorities were prevalent and well founded, as women have, in the recent past, been accused of illegal abortion or homicide after allegedly self-inducing an abortion, and several have been imprisoned [40–42].

The *Socorristas en Red (feministas que abortamos)* [Network of feminist activists supporting people who have an abortion] is a network of feminist activists who work throughout Argentina to accompany people who have medication abortions outside the formal health sector at any gestation in their pregnancy. The *Socorristas* model of “accompaniment” is described in detail elsewhere [12, 43]. In brief, the model includes in-person meetings with individuals where volunteer accompaniers, trained according to WHO protocols, provide evidence-based information on how to use abortion medications safely, and follow up by telephone to provide emotional and informational support during the use of the medications. The *Socorristas* collaborate with “friendly” health professionals, who are supportive of their work and can provide post-abortion services when needed. The *Socorristas* model of care is grounded in the understanding that everyone has the right to a safe abortion with dignity, and as such the *Socorristas* are dedicated to supporting people who seek abortion services throughout pregnancy.

Despite the existence of safe, out-of-clinic services for abortion beyond 13 weeks gestation in Argentina, medical services can also play an important role for people who self-manage their abortion, perhaps especially so for those beyond 13 weeks gestation. Due to the historic and continued restricted nature of abortion services past 14 weeks in the country, there are no published data on the number of available medical providers nationally who provide abortions after 14 weeks, but experiences from the *Socorristas* reflect a lack of providers, even in cases where the abortion would be legal [44]. For this reason, the *Socorristas* have developed several techniques to ensure that people in need of abortions beyond 13 weeks gestation have autonomy over the process and receive the information and support necessary to safely use medications for abortion. These techniques include working with health providers to ensure individuals can access post-abortion services if desired, or if complications arise, without risking their legal safety [12]. However, the *Socorristas* do not have relationships with doctors in all areas of Argentina, and the quality of the care that people receive varies by provider and facility. This study seeks to understand experiences with the health system when having abortions after 13 weeks gestation accompanied by the *Socorristas* prior to the law change in Argentina, including choices regarding healthcare-seeking and experiences with healthcare providers. This study can help understand how to improve the experiences of people who have medication abortions later in pregnancy, and how activists and health professionals can work together to provide quality services.

## Methodology

We conducted an exploratory qualitative study designed to improve understanding of women’s^1^ experiences having abortions beyond 13 weeks gestation accompanied by the *Socorristas.* [25, 45] The analysis presented here is a secondary analysis from that exploratory study. Here we focus on sections of the interviews that discussed interactions between women and health professionals during their abortion experience. We invited people from four different geographic regions of Argentina to participate: Northern Patagonia, Cuyo, Buenos Aires, and the Central region of the country. Individuals who were accompanied between June 2015 to June 2016 by the *Socorristas* for an abortion that was above 13 weeks gestation were invited to participate. Eligible participants had started their abortion with medication at home, spoke Spanish, and were 18 years or older. Individuals who were eligible to participate and had provided previous authorization to be contacted were invited to participate by the *Socorristas* who accompanied them originally. The interviews were conducted in the first half of 2016 by *Socorristas* trained to conduct interviews; the interviews were recorded after women had provided their informed consent. Given the sensitive nature of the topic, the research team, which included *Socorristas* who had years of experience accompanying, decided that in order to ensure participants felt comfortable sharing their experiences, interviewers needed to be able to quickly establish strong trust and rapport and understand the full range of interviewees’ abortion experiences. Thus we decided that the interviewers themselves would be *Socorristas*, as they would most effectively facilitate that process.

The interview guide was designed using narrative inquiry methodologies, asking participants to explain their abortion experiences from start to finish, and probing on key parts of the story [46]. The full guide was semi-structured and began asking about participants’ reproductive histories, then guided them through a narration of the decision-making about the abortion, experience of the *Socorristas* accompaniment model, the abortion itself, and experiences with the healthcare system before, during, and after their abortion. We invited 25 women to participate in the interviews; all women accepted however two later decided not to attend the interviews due to logistical reasons, resulting in a total of 23 interviews. All interviews lasted between approximately an hour and a half and two hours. The interviewers provided a snack and paid for transportation for the participant to arrive at the meeting place of the interview; all interviewees were given a book about abortion experiences as a gift to thank them for their time. The interviews were transcribed verbatim, deidentified, and the recordings were reviewed once more for confirmation. The study was approved by the Allendale Investigational Review Board.

### Analysis

This secondary analysis focused on women’s narrations of experiences with the healthcare system during their interviews. We used a content analysis approach and conducted the analysis in two cycles: the first was part of the primary analysis of interviews, where the entire study team initially read the transcripts, and used an open-coding system to identify categories inductively using what Miles, Huberman, and Saldaña (2013) describe as a holistic coding approach, identifying large chunks of text that fit into different categories that were common across interviewees [47, 48]. This first cycle of coding revealed numerous themes and categories of potential analysis. For the secondary analysis presented here, we conducted a second cycle of coding, where we only focused on women’s descriptions of their experiences with the healthcare system, and any accompanying relevant information regarding those experiences. We created a codebook that employed deductive codes defined based on the first cycle analysis and added inductive codes that arose in our re-reading of the text. The codebook was created with the objectives of being able to describe common themes in women’s experiences accessing healthcare before, during, and after their abortion. Two members of the team read through all interviews and both coded the same transcript using Atlas.ti; we then adjusted the codebook to ensure clarity and concordance, and individually coded the remaining transcripts. The study team then wrote up analytic memos describing key codes or combinations of codes and identified key themes that arose across the range of experiences. All interviews were conducted, transcribed, and analyzed in Spanish. Below we have translated relevant quotes to English which present the range of experiences and exemplary quotations describing experiences with health professionals before, during, and after the abortion.

## Results

We interviewed a total of 23 women who had abortions between 14 and 23 gestational weeks. Fifteen participants lived in urban areas, while eight lived in smaller towns. All women interviewed during the study were included in this analysis. The interviewees were between 18 and 41 years old. Thirteen had been pregnant previous to the pregnancy discussed: interviewees reported on 19 prior pregnancies total, 11 of which ended in live births, 3 in miscarriage, and 5 in abortion.

Consistent with previous literature, reasons for having an abortion beyond 13 weeks included not recognizing signs or symptoms of pregnancy until later on in their first or beginning of their second trimester, difficulties accessing abortion services with health professionals, and, for a smaller group, changes to their life circumstances motivated them to seek an abortion later in their pregnancy [17–20, 49]. Several women had multiple reasons for the delay in seeking care.

The interviews guided women to discuss the trajectory of their abortion experience, including all interactions with healthcare professionals, which included doctors, nurses, social workers in the hospital, and ultrasonographers. We identified three main time points in their experience when women had interactions with healthcare providers (before, during, and after) and how women made decisions about how to interact and share their abortion desires and experiences, as well as their experiences of treatment by those health professionals.

### Experiences seeking abortion-related services in the healthcare system

Before their abortion, most women went to a health professional to confirm the pregnancy, ask for help accessing an abortion, and/or for a dating ultrasound. Those who had made the decision to terminate the pregnancy described weighing what to say to the health professionals that they encountered, including if they wanted to ask them about abortion options.

#### Sharing the decision with a health professional

Before their abortion, almost all women went to a health professional to confirm the pregnancy, ask for help accessing an abortion, and/or to have an ultrasound to determine how many weeks along they were. Upon finding out that they were pregnant and deciding that they would terminate the pregnancy, women described how they had to decide how to act with the health professionals that they encountered during the ultrasound or clinic visit and if they wanted to ask them about abortion options. Over half of the participants spoke with a health professional about their desire to have an abortion; advice from health professionals ranged from fear tactics to straightforward referrals.

Several women described how they received negative treatment and a lack of support when they mentioned they were seeking an abortion. These women described how some providers completely rejected the idea, telling the women they couldn’t help them, that they would have to continue with the pregnancy, and scolding them for thinking about an abortion. Women also described how a few providers tried to scare them, telling them that they could encounter legal trouble, such as the quote below, or put their health at risk:

*On top of not helping me, [the doctor] made me more scared… saying ‘no, if you are going to do it you could go to prison’ and things like that. I mean, the way that [the doctor] treated me made me more scared about what I was going to do.* (23 years old, 18 weeks)

In a few cases, women reported how the health professionals who tried to dissuade them from their decision to have an abortion still provided some kind of additional information or support. In one instance, the woman described how the provider, after scolding her, gave her a prescription for medications for abortion (which ultimately didn’t work):

*[The doctor] spoke to me and kind of scolded me, “How could you not be aware, how could you not have protected yourself”. But I explained to him that he[partner] did use protection. So then he said to me, “I’m going to give you these pills,” you have to take them.* (18 years old, 14–15 weeks)

In approximately half of the cases where women shared their desires with a health professional, they were told by the providers that they couldn’t help them, but subsequently referred the woman to the *Socorristas*.

*[The gynecologist] told me that she couldn’t help me because I was too far along, that I should be careful, because I could put my life at risk. But, that’s when she mentioned your [Socorristas] name too…* (26 years old, 16 weeks)

In several other cases, when the women told a provider that they wanted an abortion, the provider supported the woman’s decision, although some women had to go to multiple providers and appointments before finding a provider who would help. When women did find a supportive provider, they described feeling extremely relieved:

*Luckily at some point they helped me, even though I thought they wouldn’t, but they helped me because they passed me your information [The Socorristas], I had never heard of the group. If the doctors hadn’t given me that information, I really don’t know what I would have done, I would have taken something through the internet and it would have been really dangerous.* (21 years old, 14 weeks)

In almost all of these cases, the providers’ support was in the form of referring them to the *Socorristas*. These cases were in regions where the *Socorristas* had local groups and a strong presence.

Several women either did not go to a healthcare provider before going to the Socorristas, or came into contact with a healthcare provider and chose not to share their abortion decision. For a few of these women, they had already been in contact with the *Socorristas* and didn’t feel it necessary to share their decision with a provider. For the rest of the women, however, they chose not to share because they were afraid of legal consequences, or because they did not think that the health professional would help, such as one woman who described her dilemma:

*You don’t know where to go, who to talk to, who can you ask [about an abortion]. You can’t ask just any doctor because they could throw you out, they could report you to the authorities.* (35 years old, 22 weeks)

#### Experiences during ultrasound

A large majority of women also visited a clinic or hospital to have an ultrasound before beginning their abortion process, as recommended by the *Socorristas*. While some women characterized the treatment they received from the person performing the ultrasound as positive or neutral, most participants described experiencing some kind of negative treatment or feeling uncomfortable during their visit. In the majority of these cases, instead of asking women if they wanted to see or hear the ultrasound, the health professional showed them images, turned the volume up to hear the cardiac activity, and in several cases even told them the sex of the fetus. Most of these women described how these experiences caused them negative feelings such as guilt or sadness:

*Yes, they showed me images…and it was awful. They showed me images. They told me the sex. And well, this was, uff! [takes a deep breath]. But I had already decided. You feel really bad, more like cruel, but I had already decided.* (27 years old, 18 weeks)

In two cases, women shared that they felt they had to go along with what the ultrasonographer did because they didn’t want anyone to suspect that they were going to have an abortion. Seeing these images, however, did not dissuade women from their decision.

Some women reported that the health professionals asked their preferences during the ultrasound, while some women pre-empted this discussion by telling the provider at the beginning they did not want to see the image. However, this did not necessarily mean that women’s preferences were respected by those health professionals. In several cases, a woman said she did not want to see the image and yet the ultrasonographer still showed it:

*I went and I asked the ultrasonographer, I said I didn’t want to see or hear it. And the man seemed like at all costs he wanted me to hear and see it. Or he wanted to tell me what the sex was, everything, everything. And I kept telling him, “no, I don’t want to”, and he would say to me, “ok, and why don’t you want to” “Because I don’t want to” I would say. And the man was saying, “if you start like that, listen, it’s going to feel like a lot of months.” And I said to myself, what part of no doesn’t he understand? He doesn’t understand “no.”* (23 years old, 21 weeks)

There were a few women who reported that they had friendly health professionals who performed the ultrasound and asked about their preferences and accepted those preferences without pressure.

### Experiences with the health system during post-abortion care

The majority of women decided to go to a hospital after taking the medication to see a medical provider. In all cases, they explained one of two reasons for this: because the *Socorristas* had suggested they go to ensure the abortion was complete, and in other cases, some women reported feeling safer and/or comfortable going to a hospital to see a doctor.

#### Weighing safety and fear when deciding whether to go to the hospital

Many women described feeling afraid to go to a hospital for fear of being treated poorly, being accused of having an abortion, and/or reported to the authorities. While some participants reported wanting to go to a hospital because they felt it would be better for their physical health, many of these same women were concerned about their abortions being discovered. This preference for going to the hospital was more prevalent in cases where the *Socorristas* could help arrange the visit so that the woman could go to the hospital when there was a friendly, supportive provider on call:

*They had already told me who would be at the hospital, at what time I should go to the hospital according to the time that this doctor would be starting, we calculated the timing of when I should take the pills so that I would arrive when he was there…for me it was better that he was there, I felt more calm that way.* (35 years old, 22 weeks)

In cases where it was safe to do so, the women described how the *Socorristas* advised women that, if they wanted to, they could share with a health professional that they had taken medication, because those providers would not put the woman in legal danger.

In other cases, the fear that someone in the health system could find out about the abortion and report it to the authorities or treat them poorly did not subside even with the *Socorristas*’ assurances. One woman chose not to share anything in the hospital because she had to hide the abortion from her partner, who emotionally and physically abused her:

*I was afraid that…for example, if I went there and a doctor found out about this…for example, that they examined me and said, “No, she tried to have an abortion.” This was my fear, I was so afraid that this would really happen. Because I said to myself, “I will die if they say that I tried to have an abortion, because he [partner] would kill me. This was my fear.”* (32 years old, 23 weeks)

A smaller group of women did not go to the hospital after taking abortion medications. For two women, the fear and uncertainty about what could happen in the hospital was enough to encourage them not to go:

*No why, so that they treat me poorly or something like that? No, forget about it. No, no because with the fuss that would be made, it would be a mess…I never felt that bad, the pain that I felt, I mean, it was like very, very strong cramps, but no more than this.* (23 years old, 21 weeks)

Finally, several women didn’t seek support from a health professional either because the abortion happened quickly and they didn’t have time to go to a health facility, and/or they believed it wasn’t necessary in that moment. Among this group, all women had abortions between 14 and 16 weeks; the majority had been pregnant before and two had had an abortion before and said they had felt prepared for what would happen this time around.

#### Experiences with health professionals in the hospital

The majority of women who went to a hospital after taking the medication, often when they were still showing signs of an abortion, reported some mistreatment from a health professional—including receiving judgmental/stigmatizing care, or inappropriate treatment for the situation. While some women were clear that the mistreatment stemmed from a health professional suspecting that they had self-induced an abortion, in other cases the reasons for mistreatment were less clear.

When asked directly, or even pressured by health professionals, over half of participants who went to a hospital chose not to discuss their use of abortion medication. Most commonly, women said they did not feel comfortable discussing their use of medications to induce abortion and were afraid of the consequences of doing so:

*And there she [health professional] came in to bother me, “did you take anything?” And to my mother, “Did she take anything”. But awful, always awful, never saying it like, “ok, if you took something, tell me so I can help you, tell me what you took.” Instead, always treating me poorly, scolding me, “No, no,” I kept saying, “no.”* (35 years old, 22 weeks)

Three of these women, all of whom reported interacting with health providers who suspected them of having induced an abortion, also reported receiving painful and/or unnecessary treatment and reported that they thought it was attributed to the suspicions of the provider.

Participants also reported cases of poor medical care that they attributed to providers not being properly prepared to manage their case. In several cases, when women first went to a hospital showing symptoms of an abortion, such as bleeding or cramping, the providers determined these women did not need care, or didn’t believe they were pregnant. Most of these women were sent home and ended up finishing their abortion at home without the medical attention they had been seeking. One woman was in the process of her abortion when she went to a clinic but the clinicians told her she had to go to a lab for a blood test for the pregnancy before they could provide any additional care. Afterwards, they wanted to send her home:

*Those stupid people [referring to the doctors], I was telling them I was pregnant, that it hurt, that they look at me, at something (because I thought I was close to expelling, I wanted them to see if they could do something), and they gave me buscapina [pain reliever] and told me to go home to bed. Imagine if I had wanted this baby?! Those stupid people would have made me lose the baby!* (37 years old, 20 weeks)

In three cases where women did not feel comfortable revealing their use of abortion medications to healthcare providers, they were transferred to other hospitals because the providers, thinking that the women wanted to continue the pregnancy, believed they needed a higher level of care (such as a neonatology unit). In several additional cases where women were afraid to disclose their abortion, providers assumed that women wanted to continue the pregnancies and placed them in rooms with other women who were in the process of giving birth. As a result of non-disclosure, the justifiable actions of health professionals caused stress and anguish among the women having abortions. For example, one participant was in a hospital for three days while providers tried to maintain the pregnancy:

*They would come and do tests on me, I heard the heart beat for three days in a row. It was really hard.* (24 years old, 18 weeks)

Women also spoke of positive experiences with some health professionals, where the care they received was supportive and judgement-free. One woman describes her response when a provider asked her if she had taken anything:

*I started to cry, I thought, I don’t know, that they would treat me badly, verbally, lots of things go through your head… what about the police…when it’s so difficult you imagine lots of things, your brain is working in overtime. And I don’t know, I started crying and told them the truth. They didn’t ask me anything in that moment about it, not what it was, or with whom, they just said, “Ok, we don’t judge. Only the three of us and you will know.”* (27 years old, 18 weeks)

While over half of participants who went to hospitals during their abortion did not disclose their use of medications, the other  half of participants did disclose to at least one provider. Several participants who were asked directly by health professionals about use of medications for abortion chose to disclose their abortion. Even then, however, some women expressed some concern with sharing the information:

*Well, so I told them [paramedics who came with an ambulance] that yes, I had used the medication… “I’m not sure, I’m a little afraid that the police will show up to interrogate me,” I said, “What if they put me in prison or something?” And [the paramedics] said to me: “No, we are not going to bring you to the police or anything, we want to know because we have to tell the doctor. We will leave you in the hospital and we have to tell the doctor what happened. It’s a secret amongst us.”* (21 years old, 19 weeks)

In a few cases, women reported that providers were ready to receive them at the hospital due to prior contact between the *Socorristas*, who had let them know that the woman would be going to the hospital after taking the medication. In these cases the doctors simply attended to them directly, without asking questions:

*Well, when I arrived at the hospital I was having a lot of contractions, and they attended to me immediately. When I was admitted there was [the doctor the Socorristas had notified], with another doctor, a woman, the two of them… it was clear they already knew something because they admitted me immediately, and didn’t ask many questions, it was clear they were already aware of what was going on.* (21 years old, 14 weeks)

Though the participants often did not know whether the health professionals were considered ‘friendly’ by the *Socorristas*, all participants who reported feeling supported by a hospital-based health professionals lived in areas where the *Socorristas* have strong collectives and have developed relationships with health professionals. While not every provider that these participants interacted with was supportive, based on the experiences they described, most of the supportive providers they came into contact with were identified by the research team during the interviews as part of the *Socorristas’* extended network of “friendly” health professionals.

## Discussion

This qualitative study demonstrates experiences of women who have medication abortions outside the formal health system beyond 13 weeks, and their experiences with the health sector. Almost all women in this study interacted with a health professional during their abortion process while accompanied by the *Socorristas en Red,* demonstrating the potential for pregnant people to encounter various models of care (in this case, activists and health professionals) during their experience with self-managed medication abortion.

Just as we have seen in other studies where abortion is restricted, these women encountered various barriers to abortion services; for some, trying to find someone to help them opened them up to criticisms and judgement from health professionals who were against their decision, and delayed their care [18, 35, 36]. Yet women went to providers for an array of reasons, both before, during, and after their abortion. For a range of reasons, and despite feeling well-supported by the accompaniment group, some participants reported that they preferred to go to a hospital during their abortion process for their own perceived safety and comfort, and in some cases this was facilitated by relationships that the *Socorristas* have developed with local health professionals. Two other studies of people self-managing their abortions also observed healthcare-seeking behaviors during abortion, and found that many chose to go to a provider after their abortion to confirm completion [14, 50]. This is one reason to access a provider during a self-managed abortion, and indeed we see that in the case of abortions beyond 13 weeks, the interactions with providers were more frequent.

At the same time, some women in our study reported being concerned about legal repercussions if health professionals found out they had taken medications for abortion, and tried to avoid the health system as much as possible. These fears of legal repercussions are not uncommon; indeed, even in countries such as Colombia, where abortion was available under a wide array of exceptions prior to 2022 when abortion was decriminalized, participants in other studies have reported fears of legal repercussions delaying or impeding their care [51–53]. In this study many women did not receive quality care because they felt they could not share that they had taken medications with the doctor. This is seen in many stages of their abortion process; the poor care occurred in some instances because the doctors suspected the woman of an abortion or wanting an abortion and treated them poorly for their choice, while in other cases women felt they could not share they were having an abortion in the hospital, and were instead treated to try to maintain the pregnancy. These findings align with two previous studies which have identified poor care of people who self-managed their abortions in Argentina, demonstrating the resistance of some providers to provide quality care due to their personal judgements [40, 41].

Though these experiences of poor treatment exist, in our study some women also had very positive experiences with supportive health professionals. Given the health professionals’ close ties with the *Socorristas*, as reported by the study participants, it is probable that many of these health professionals were part of the National Network of Health Professionals for the Right to Choose; this network has been described elsewhere, and has been a powerful force to ensure providers who see people seeking abortion services are supported [54, 55]. Indeed, it is likely these providers, because of their involvement in an organization that seeks to destigmatize abortion, also provide much more supportive and accepting care to people seeking abortion services.

In our study, links between activists and the formal health system operated bi-directionally, in that women reported that providers often referred them to the *Socorristas* when they could not or did not want to provide care, and, where the *Socorristas* have strong networks of “friendly” providers, women were often linked to providers who would support them during and after their abortions. In these cases, women tended to report better experiences in the healthcare system. Several studies conducted with abortion accompaniment groups demonstrate a high level of satisfaction with the services offered, and one other study has documented the ways in which people having abortions in restrictive legal settings interact with both clinical and community-based services during their abortions [12, 15, 31, 50]. This is the first study, to our knowledge, that demonstrates how women who self-manage abortions beyond 13 weeks with the support of abortion accompaniers also interact with providers in the clinical health system to receive the care they need and want in restrictive settings. This model of care, operating in a restrictive legal setting, draws on the strengths of clinical and community-based systems and results in person-centered and high-quality abortion care.

### Limitations

The nature of qualitative work is to examine a population and experience in depth, however we cannot generalize findings about this study; those who have abortions in other contexts inside and outside of Argentina may have different experiences, especially where abortion is more severely criminalized and/or stigmatized. Additionally, the experiences described here include those of women accessing the healthcare system while also having support from abortion accompaniers; it is likely that those who access the healthcare system through other means, or who identify as a different gender, have different healthcare experiences that should be illuminated in future research.

Since these data were collected in 2016, abortion legalization was debated in two rounds of Congress in Argentina, in 2018 and 2020, and legalized through 14 weeks in the final round. In addition to the legal changes, reports and anecdotes suggest that there has been a certain amount of “destigmatization” around abortion nationally which may impact how society, and health professionals specifically, view abortion, including abortion after 14 weeks given that it continues to only be legal in certain circumstances to date [44, 56, 57]. Though the experiences here may not reflect in its whole the current nature of abortion access in Argentina, globally, abortions later in pregnancy are far more stigmatized than first-trimester, and anecdotes from after the law in Argentina have continued to show challenges with abortion access after 14 weeks. [58, 59]

### Recommendations

Though abortion through 14 weeks is now legal without exception in Argentina, a history that includes maltreatment and at times criminalization of people who self-manage their abortions may still dissuade those seeking abortions from being transparent with health professionals in cases of self-managed abortion. This context is not unique; globally, when abortion is criminalized, legal fears lead to barriers to access [18, 51, 52, 60]. In addition to decriminalizing abortion to improve access and quality, health professionals should be trained in how to provide quality pre- and post-abortion care, their obligations to their patients during this time, and how to tactfully broach the subject to ensure quality care in any circumstance. Further research to document the experiences of people seeking healthcare in Argentina during the course of their abortion could systematically identify areas for training, and regions in need of training, to improve care for these individuals when accessing the medical systems.

With the increasing use of medications for abortion, specifically self-managed abortion, the model of care and interdisciplinary collaboration between activists and health professionals demonstrated here may also be a useful example for high-quality, person-centered abortion provision in both restrictive and less restrictive settings. Such a model could serve as an example for other contexts where activists and “friendly” health professionals share a common goal for those who need abortions—perhaps especially those happening beyond 13 weeks gestation—to have a safe, high-quality, person-centered abortion experience regardless of the setting. Further research to understand this model, people’s preferences when seeking abortion beyond 13 weeks using medication abortion, and how to assess and meet those preferences and ensure safety, could lead to expanded access to high-quality abortion care tailored to individuals to ensure they can access that care when and how they prefer.

## Conclusions

In our study, we found that women self-managing abortions past 13 weeks sought healthcare from a variety of providers, even when self-managing their abortion accompanied by feminist activists, though the treatment by health professionals varied from very negative to extremely positive and supportive. The experiences of women who went to health professionals with established relationships with the feminist activist network, the *Socorristas*, appeared to be more positive than those who did not. Quality abortion care past 13 weeks can be improved through decriminalization of abortion, fomenting additional relationships between health professionals and community-based activists supporting people who are self-managing their abortions, and training health professionals to support people’s decision making about abortion and properly assess and support people who seek abortion-related services.

## Data Availability

Portions of the data that support the findings of this study are available on reasonable request from the corresponding author, BKO. The full dataset is not publicly available due to their containing information that could compromise the privacy of research participants.

## Abbreviation

WHO: World Health Organization

## Footnotes

1 We refer in this article to ‘people’ who seek abortions because not all people who can carry a pregnancy identify as women. However, in our study, during the course of their interviews, all participants identified as women, and thus we refer to them as such when we discuss our study population.
